# Comparative effectiveness of arthroscopic-assisted versus open reduction and internal fixation in the treatment of tibial plateau fractures: a retrospective study

**DOI:** 10.1186/s40001-026-03946-4

**Published:** 2026-01-26

**Authors:** Zhizhong Tian, Ruiping Jia, Xinjun Guo, Likai Zhang, Xiao Yun, Lin Wang

**Affiliations:** Department of Orthopedics, Xinxiang Central Hospital, The Fourth Clinical College of Xinxiang Medical University, No.56 Jinsui Avenue, Weibin District, Xinxiang, 453000 Henan Province China

**Keywords:** Tibial plateau fractures, Arthroscopic-assisted reduction and internal fixation (ARIF), Open reduction and internal fixation, Surgical outcomes

## Abstract

**Background:**

Tibial plateau fractures require accurate articular reduction and stable fixation to restore knee function and minimize complications. Arthroscopic-assisted reduction and internal fixation (ARIF) may reduce soft-tissue disruption compared with open reduction and internal fixation (ORIF). To compare clinical outcomes and complication profiles of ARIF versus ORIF for tibial plateau fractures.

**Methods:**

This retrospective cohort study included adults (≥ 18 years) with radiographically confirmed tibial plateau fractures treated surgically between January 2020 and January 2024 at a single center. Patients underwent ORIF (traditional group, n = 65) or ARIF (arthroscopic-assisted group, n = 68). Outcomes included intraoperative blood loss, operative time, incision length, time to ambulation, fracture healing time, wound healing time, knee range of motion (ROM), pain assessed by the Visual Analogue Scale (VAS), Lysholm score categories (excellent/good/fair/poor) with effectiveness rate (excellent + good), and postoperative complications. Level of evidence: III.

**Results:**

Baseline characteristics were comparable (age: 38.1 ± 4.3 vs 37.9 ± 4.1 years, p = 0.748; sex distribution, p = 0.514). Compared with ORIF, ARIF was associated with lower blood loss (200 ± 28 vs 250 ± 50 mL; t = 4.779; p < 0.05), shorter operative time (73 ± 12 vs 90 ± 13 min; t = 5.263; p < 0.05), and smaller incisions (6.8 ± 1.3 vs 13.8 ± 2.3 cm; t = 14.512; p < 0.05). ARIF showed faster recovery, including earlier ambulation (3.2 ± 0.7 vs 5.2 ± 1.6 days; t = 6.273; p < 0.05) and shorter fracture healing time (81.3 ± 2.3 vs 87.1 ± 2.8 days; t = 8.767; p < 0.05). At 6 months, ARIF achieved greater knee flexion (108.0 ± 9.0 vs 97.0 ± 9.6°; t = 4.579; p < 0.05) and lower VAS pain (0.9 ± 0.3 vs 1.4 ± 0.4; t = 5.477; p < 0.05). The Lysholm-based effectiveness rate was higher with ARIF (85.3% vs 66.2%; χ^2^ = 5.657; p < 0.05). Traumatic arthritis and wound infection were less frequent after ARIF (4.4% vs 17.0%, χ^2^ = 4.275, p < 0.05; 5.9% vs 20.0%, χ^2^ = 4.743, p < 0.05).

**Conclusions:**

In this retrospective cohort, ARIF was associated with improved perioperative efficiency, faster recovery, better short-term functional outcomes, and fewer selected complications compared with ORIF. Prospective, multicenter randomized studies with longer follow-up are warranted to confirm these findings and to further clarify the long-term functional outcomes and complication profiles of ARIF compared with ORIF.

## Introduction

Tibial plateau fractures are complex intra-articular injuries of the proximal tibia, commonly caused by high-energy mechanisms such as motor vehicle collisions or falls from height. They pose substantial challenges to orthopedic surgeons because of articular surface disruption, concomitant soft-tissue injury, and the risk of persistent functional limitation and post-traumatic sequelae [1–3]. Traditionally, open reduction and internal fixation (ORIF) has been considered the standard surgical approach, enabling direct exposure of the fracture site, anatomic reduction, and stable fixation using plates, screws, and, when indicated, external fixation [4–6]. However, ORIF may require extensive soft-tissue dissection and periosteal stripping, which can compromise local vascularity and increase the risk of wound-related complications, including infection and nonunion [7].

In recent years, arthroscopic-assisted reduction and internal fixation (ARIF) has emerged as an alternative strategy for tibial plateau fractures. Arthroscopy provides minimally invasive access to the joint, superior visualization of intra-articular anatomy, and the opportunity to identify and manage concomitant meniscal, chondral, and ligamentous injuries during the same procedure [8, 9]. With arthroscopic guidance, fracture reduction and fixation can be performed with reduced soft-tissue trauma and improved preservation of regional blood supply [10, 11]. Accordingly, ARIF has been proposed to decrease the morbidity associated with traditional ORIF, particularly in patients with vulnerable soft tissues, while supporting earlier rehabilitation and potentially lowering postoperative complication rates [12, 13]. While previous studies comparing ARIF with traditional ORIF have reported on outcomes such as functional recovery, complication rates, and surgical efficiency, a key gap remains in the detailed assessment of intraoperative variables and recovery metrics across a broader range of tibial plateau fracture types [14]. Most existing studies have either focused on specific fracture types or have not comprehensively compared a wider range of surgical parameters and recovery times, limiting their ability to fully capture the potential advantages of ARIF in clinical practice [14, 15].

To address this gap, our study provides a comprehensive analysis by including a diverse cohort of patients with Schatzker types I-IV fractures. We also evaluate detailed intraoperative and postoperative recovery metrics, including incision length, intraoperative blood loss, and time to ambulation. By examining these additional variables, as well as complications like traumatic arthritis and wound infections, our study offers new insights into the broader clinical advantages of ARIF. The objective of this study is to compare the clinical outcomes and complications associated with ARIF versus ORIF in the management of tibial plateau fractures. Through this comparative analysis, we aim to identify the clinical benefits, recovery differences, and complication rates associated with ARIF, thereby offering new insights into its potential as a less invasive alternative to traditional ORIF. Ultimately, our findings seek to contribute to the ongoing discourse surrounding the optimal surgical management of tibial plateau fractures and inform clinical decision-making practices.

## Methods

### Study design

A retrospective cohort study was conducted at our institution to compare the effectiveness of arthroscopic-assisted reduction and internal fixation with conventional open reduction and internal fixation in the treatment of tibial plateau fractures. The study period extended from January 2020 to January 2024. Patients treated with open reduction and internal fixation were assigned to the conventional surgery group, whereas those treated with arthroscopic-assisted reduction and internal fixation were assigned to the arthroscopic-assisted group. All procedures were performed by the same orthopedic trauma team, whose surgeons had formal training and over 8 years of experience with both surgical techniques for tibial plateau fractures. The study protocol was reviewed and approved by the institutional ethics committee. All procedures were conducted in accordance with relevant guidelines and regulations and adhered to the principles of the Declaration of Helsinki. Patient data were handled confidentially, and all personal identifiers were removed prior to analysis to ensure privacy.

### Inclusion and exclusion criteria

Inclusion Criteria:

1. Age and Sex: Patients aged 18 years or older, irrespective of sex.
2. Diagnosis: Radiographically confirmed tibial plateau fractures based on X-ray and computed tomography (CT) imaging.
3. Treatment Modality: Patients who underwent surgical management for tibial plateau fractures using either ORIF or arthroscopic-assisted reduction and internal fixation during the study period.
4. Informed Consent: Patients who provided written informed consent permitting the use of their clinical data for research purposes.
5. Absence of Severe Complications: Patients without major vascular or nerve injuries and without evidence of compartment syndrome.

Exclusion Criteria:

1. Previous Knee Surgery: History of prior knee surgery or fractures involving the same knee.
2. Polytrauma: Presence of multiple traumatic injuries in which tibial plateau fracture was not the primary injury.
3. Chronic Joint Disease: Coexisting chronic inflammatory joint diseases, such as rheumatoid arthritis, or other conditions that could adversely affect bone quality or fracture healing.
4. Non-surgical Treatment: Patients managed conservatively without surgical intervention.
5. Incomplete Data: Incomplete medical records or missing follow-up information.
6. Open Fractures: Patients presenting with open tibial plateau fractures.

### Surgical technique for traditional open reduction and internal fixation of tibial plateau fractures

Following induction of anesthesia, patients were positioned supine on the operating table. The operative field was prepared and draped in a sterile fashion, and a pneumatic tourniquet was applied at a pressure of 60 kPa to control intraoperative bleeding. Surgical exposure was achieved through medial or lateral incisions according to fracture location and complexity. In selected cases, a Y-shaped incision was used to provide wider access to the joint cavity when necessary. The surgical approach was selected to ensure adequate visualization and management of the articular surface and associated structures. After joint exposure, a comprehensive assessment of intra-articular structures was performed, including evaluation of the menisci and cruciate ligaments. Partially injured collateral ligaments were stabilized as needed. In cases of complete cruciate ligament rupture, staged repair or reconstruction was performed to restore knee stability and function.

Fixation strategies were determined according to the Schatzker fracture classification. Schatzker type I fractures were typically treated with two or three cannulated screws to achieve stable fixation. Schatzker type II and III fractures commonly required T-shaped buttress plate fixation to support the articular surface and maintain alignment. Schatzker type IV fractures were generally managed with dual plating techniques to provide sufficient mechanical stability and promote optimal fracture healing.

### Surgical technique for arthroscopic-assisted reduction and internal fixation of tibial plateau fractures technique

Following induction of anesthesia, patients were positioned supine, and the operative field was prepared using standard sterile techniques. Arthroscopic evaluation was performed to assess intra-articular hematoma and the extent of articular surface involvement. Kirschner wires and traction devices were placed parallel to the tibial shaft and calcaneal tubercle to facilitate controlled reduction. An anterolateral portal was established for arthroscopic access. Hematoma evacuation was performed initially to optimize visualization of the fracture site. Fracture alignment was confirmed arthroscopically, and traction was applied to reduce displaced fragments. Adjustments to the traction device were made as needed to correct lateral displacement. Once satisfactory reduction was achieved, arthroscopic inspection was used to confirm articular congruity and to address residual depression.

Fixation strategies were tailored according to the Schatzker classification. Schatzker type I fractures without displacement were stabilized using one or two cancellous screws; if displacement was present, traction-assisted reduction was performed prior to fixation. Schatzker type II fractures, characterized by split-depression patterns in weight-bearing regions, required precise localization of the depressed fragment using an anterior cruciate ligament locator, followed by elevation with a cannulated impactor. Bone grafting was performed, and the articular surface was anatomically restored using autologous iliac bone support. Management of Schatzker type III fractures followed a similar approach, involving elevation of the depressed area with subsequent minimally invasive fixation and bone grafting. For Schatzker type IV fractures, arthroscopic-assisted traction reduction was performed and secured with cannulated cancellous screws, with supplemental plating applied when additional stability was required.

### Data collection and variables examined

Given the retrospective nature of the study, patients were grouped according to the surgical technique documented in the electronic medical records. Outcome assessments were based on predefined clinical and radiological criteria and were performed by assessors who were not involved in the surgical procedures. Postoperative rehabilitation was standardized across both groups and followed the same institutional protocol [16, 17]. The protocol was formulated in accordance with internationally recommended postoperative management principles for tibial plateau fractures and implemented uniformly at our institution, including weight-bearing progression, range-of-motion exercises, and physiotherapy programs.

Surgical-related indices: Intraoperative parameters, including blood loss, operative time, incision length, and time to postoperative ambulation, fracture healing, and wound healing, were systematically recorded.

Fracture healing was defined by the absence of percussion pain and local tenderness at the fracture site, restoration of functional activity, radiographic evidence of blurred fracture lines with continuous callus formation, and the ability to ambulate more than 30 steps independently on a flat surface for approximately 3 min without deformity during a 2-week observation period.

Pain intensity: Pain was assessed using the Visual Analogue Scale (VAS) preoperatively and at 6 months postoperatively. The VAS ranges from 0, indicating no pain, to 10, indicating the worst imaginable pain, with higher scores reflecting greater pain severity.

Range of motion of the knee joint: Knee joint function, including flexion and extension, was measured using a goniometric brace board. Assessments were performed preoperatively and at 6 months postoperatively to evaluate functional recovery.

Efficacy evaluation: Knee function was evaluated at 6 months postoperatively using the Lysholm scoring system. Scores were categorized as excellent (> 90), good (75–89), fair (60–74), or poor (< 59). The effectiveness rate was calculated as the proportion of patients achieving excellent or good outcomes relative to the total number of cases.

Complications: Postoperative complications, including post-traumatic arthritis and wound infection, were assessed based on patient-reported symptoms, routine laboratory examinations, and follow-up imaging studies, such as radiography or computed tomography.

### Statistical analysis

Statistical analyses were performed using SPSS software, version 27.0. Data were categorized as either quantitative or categorical variables. The distribution of quantitative variables was assessed using normality tests. For normally distributed data, results were expressed as means ± standard deviations and compared using independent samples t-tests. Non-normally distributed data were reported as medians and interquartile ranges [M (P25, P75)], and comparisons between groups were made using the Mann–Whitney U test. Categorical variables were presented as frequencies and percentages. The Chi-square (χ^2^) test was employed to assess associations between categorical variables. All statistical tests were two-sided, with a significance threshold set at p < 0.05.

## Results

### Patient demographics and clinical characteristics

**Fig. 1:**
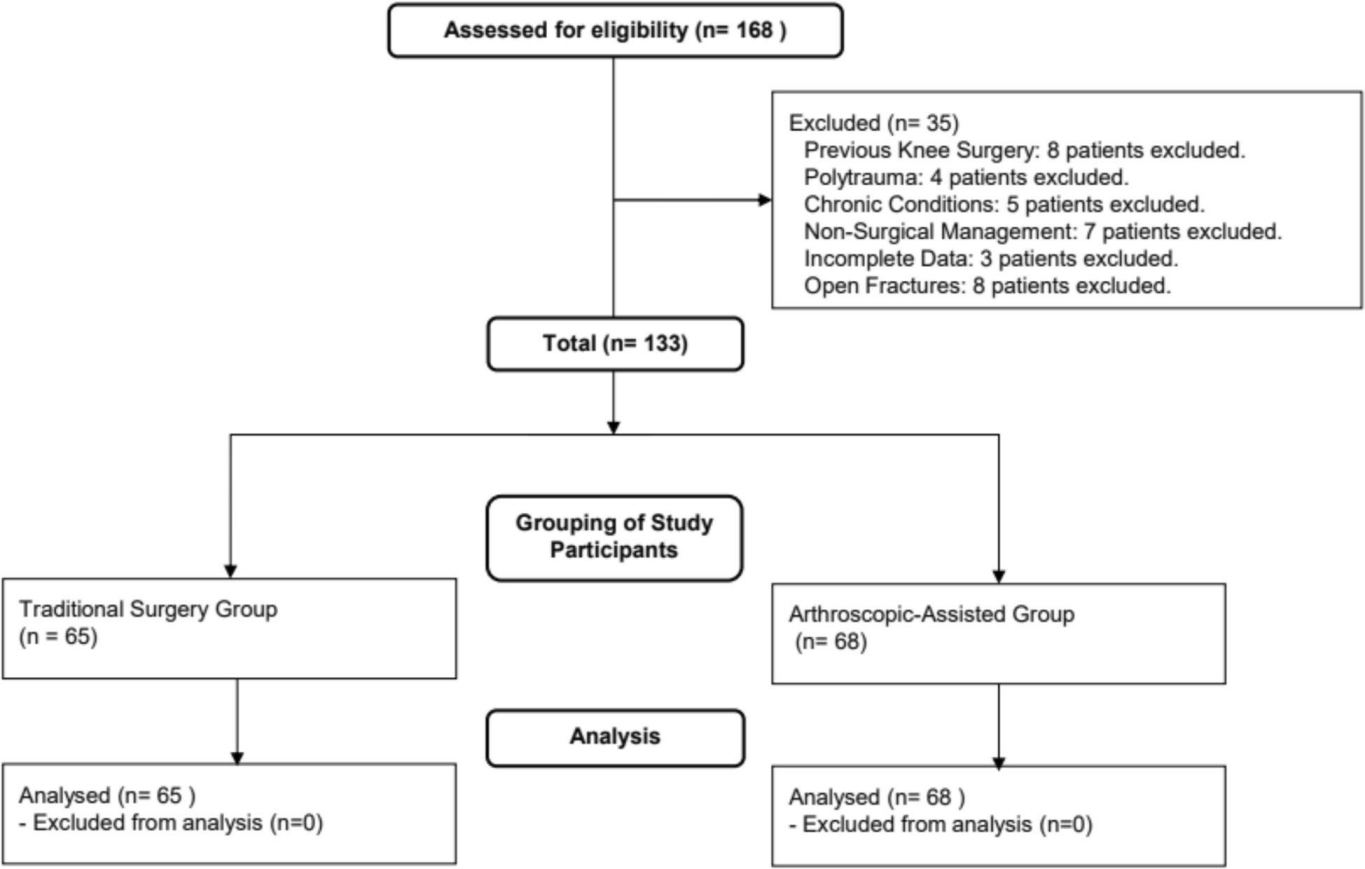
Study participant flowchart

The study compared 65 patients in the traditional surgery group and 68 in the arthroscopic-assisted group (Fig. 1). No significant differences were observed in terms of age (mean ± SD: 38.1 ± 4.3 years vs. 37.9 ± 4.1 years; t = 0.326, p = 0.748) or gender distribution (M/F: 32/33 vs. 34/34; χ^2^ = 1.625, p = 0.514). Both groups had similar disease durations prior to surgery (3.5 ± 0.9 days vs. 3.4 ± 0.8 days; t = 0.529, p = 0.441), and comparable rates of diabetes (15 vs. 14; χ^2^ = 1.934, p = 0.842). The causes of injury, car accidents, falls, and heavy objects, were similarly distributed between groups, as were the sides of the injuries and fracture types according to the Schatzker classification. No statistically significant differences were observed in these variables (Table 1). These findings indicate that both groups had comparable demographic and clinical characteristics at baseline.

**Table 1:** Demographic and clinical characteristics of patients undergoing traditional surgery and arthroscopic-assisted surgery for tibial plateau fractures

Characteristics	Traditional surgery group (n = 65)	Arthroscopic-assisted group (n = 68)	Statistical values
Age (x ± s, years)	38.1 ± 4.3	37.9 ± 4.1	t = 0.326, p = 0.748
Sex (M/F, n)	32/33	34/34	χ^2^ = 1.625, p = 0.514
Disease duration (x ± s, days)	3.5 ± 0.9	3.4 ± 0.8	t = 0.529, p = 0.441
Diabetes (n)	Yes: 15, No: 50	Yes: 14, No: 54	χ^2^ = 1.934, p = 0.842
Injury cause (n)	Car accident: 33, Fall: 17, Heavy object: 15	Car accident: 32, Fall: 16, Heavy object: 20	p > 0.05
Injury side (n)	Left: 30, Right: 35	Left: 31, Right: 37	p > 0.05
Fracture type (n)	Schatzker I: 10, II: 13, III: 27, IV: 15	Schatzker I: 9, II: 12, III: 28, IV: 19	p > 0.05
Treatment Methods (Cannulated Screws/Plates)	Schatzker I: 5/5, II: 7/6, III: 15/12, IV: 8/7	Schatzker I: 4/5, II: 6/6, III: 14/14, IV: 9/10	p > 0.05
Follow-up duration (x ± s, months)	15.9 ± 2.2	16.1 ± 2.0	p > 0.05

*M/F* male/female, *x* ± *s* mean ± standard deviation, *n* number of patients, *Schatzker I–IV* Schatzker classification of tibial plateau fractures

### Surgical metrics outcomes of two surgical techniques for tibial plateau fractures

This study evaluated surgical metrics between traditional surgery and ARIF in the treatment of tibial plateau fractures. Significant improvements in surgical efficiency and recovery outcomes were observed with the arthroscopic-assisted approach. Patients in the arthroscopic-assisted group experienced less intraoperative blood loss (200 ± 28 ml) compared to the traditional surgery group (250 ± 50 ml; t = 4.779, p < 0.05). The arthroscopic-assisted technique was also associated with shorter surgery durations (73 ± 12 min vs. 90 ± 13 min; t = 5.263, p < 0.05) and significantly reduced incision lengths (6.8 ± 1.3 cm vs. 13.8 ± 2.3 cm; t = 14.512, p < 0.05). Recovery metrics were also favorable in the arthroscopic-assisted group, with a quicker time to ambulation (3.2 ± 0.7 days vs. 5.2 ± 1.6 days; t = 6.273, p < 0.05) and faster fracture healing times (81.3 ± 2.3 days vs. 87.1 ± 2.8 days; t = 8.767, p < 0.05). Additionally, wound healing was more rapid in the arthroscopic-assisted group (9.0 ± 1.2 days vs. 13.5 ± 1.6 days; t = 12.324, p < 0.05) (Table 2).

**Table 2:** Comparative analysis of surgical metrics in traditional surgery vs. Arthroscopic-assisted reduction and internal fixation (ARIF) for tibial plateau fractures

Metrics	Traditional surgery group (n = 65)	Arthroscopic-assisted group (n = 68)	t-value	Mean difference	95% CI	p-value
Intraoperative blood loss (ml)	250 ± 50	200 ± 28	4.779	− 50.0	− 63.7 to − 36.3	< 0.05
Surgery duration (min)	90 ± 13	73 ± 12	5.263	− 17.0	− 21.9 to − 12.1	< 0.05
Incision length (cm)	13.8 ± 2.3	6.8 ± 1.3	14.512	− 7.0	− 7.6 to − 6.4	< 0.05
Time to ambulation (d)	5.2 ± 1.6	3.2 ± 0.7	6.273	− 2.0	− 2.4 to − 1.6	< 0.05
Fracture healing time (d)	87.1 ± 2.8	81.3 ± 2.3	8.767	− 5.8	− 6.7 to − 4.9	< 0.05
Wound healing time(time to suture removal) (d)	13.5 ± 1.6	9.0 ± 1.2	12.324	− 4.5	− 5.0 to − 4.0	< 0.05

*n* number of patients, *ml* milliliters, *min* minutes, *cm* centimeters, *d* days, *MD* mean difference (arthroscopic-assisted minus traditional surgery), *CI* 95% confidence interval

### Joint mobility and pain outcomes of two surgical techniques for tibial plateau fractures

Initially, both groups demonstrated similar knee extension and flexion capabilities and reported comparable pain levels, as measured by the VAS. Specifically, preoperative knee extension was nearly identical between the traditional (− 1.6 ± 0.6 degrees) and arthroscopic-assisted groups (− 1.5 ± 0.5 degrees; t = 0.701, p = 0.486). Knee flexion prior to surgery also showed no significant difference between the traditional (66.3 ± 7.3 degrees) and arthroscopic-assisted groups (68.3 ± 7.5 degrees; t = 1.047, p = 0.300). Additionally, the preoperative VAS scores were closely matched (6.8 ± 1.1 vs. 6.7 ± 1.2; t = 0.336, p = 0.738). At 6 months postoperatively, notable improvements were observed in the arthroscopic-assisted group. Postoperative knee extension improved significantly in the arthroscopic-assisted group (2.5 ± 0.7 degrees) compared to the traditional group (1.9 ± 0.7 degrees; t = 3.320, p < 0.05). Knee flexion also showed superior enhancement, with the arthroscopic-assisted group reaching 108.0 ± 9.0 degrees compared to 97.0 ± 9.6 degrees in the traditional group (t = 4.579, p < 0.05). Furthermore, postoperative VAS scores indicated significantly less pain in the arthroscopic-assisted group (0.9 ± 0.3) versus the traditional group (1.4 ± 0.4; t = 5.477, p < 0.05) (Table 3).

**Table 3:** Preoperative and 6-month postoperative clinical outcomes in traditional surgery vs. Arthroscopic-assisted reduction and internal fixation (ARIF) for tibial plateau fractures

Outcome measures	Measurement time	Traditional surgery group (n = 65)	Arthroscopic-assisted group (n = 68)	Mean difference	95% CI	t-value	p-value
Knee extension (°)	Preoperative	−1.6 ± 0.6	−1.5 ± 0.5	0.1	− 0.1 to 0.3	0.701	0.486
Knee flexion (°)	Preoperative	66.3 ± 7.3	68.3 ± 7.5	2	− 0.6 to 4.6	1.047	0.300
VAS score	Preoperative	6.8 ± 1.1	6.7 ± 1.2	− 0.1	− 0.5 to 0.3	0.336	0.738
Knee extension (°)	6 months postoperative	1.9 ± 0.7	2.5 ± 0.7	0.6	0.3–0.9	3.320	< 0.05
Knee flexion (°)	6 months postoperative	97.0 ± 9.6	108.0 ± 9.0	11	7.2–14.8	4.579	< 0.05
VAS score	6 months postoperative	1.4 ± 0.4	0.9 ± 0.3	− 0.5	− 0.6 to − 0.4	5.477	< 0.05

*n* number of patients, *VAS* Visual Analogue Scale, *MD (Mean Difference)* difference between the arthroscopic-assisted group and the traditional surgery group, *CI* confidence interval, ° degrees

### Comparative effectiveness of surgical techniques for tibial plateau fractures

This analysis compared the overall clinical efficacy of traditional ORIF versus ARIF for the treatment of tibial plateau fractures, based on patient outcomes classified as excellent, good, fair, or poor. At final follow-up (mean follow-up duration: 16.1 ± 2.0 months in the arthroscopic-assisted group and 15.9 ± 2.2 months in the traditional surgery group), the ARIF group demonstrated a higher proportion of excellent outcomes, with 30 of 68 patients (44.1%) achieving this classification, compared to 19 of 65 patients (29.2%) in the ORIF group. Additionally, good outcomes were reported in 28 patients (41.2%) in the ARIF group and 24 patients (36.9%) in the ORIF group. The ARIF group had fewer patients with fair (4.4%) or poor outcomes (10.3%) than the ORIF group, in which 9 (13.8%) and 13 (20.0%) patients, respectively, fell into these categories. The overall effectiveness rate, defined as the proportion of patients rated as excellent or good, was significantly higher in the ARIF group (85.3%, 58 of 68) compared to the ORIF group (66.2%, 43 of 65), with a Chi-square statistic of 5.657 (p < 0.05; Table 4). These findings indicate that ARIF is associated with superior short-term clinical outcomes in the surgical management of tibial plateau fractures.

**Table 4:** Treatment effectiveness based on Lysholm score in traditional surgery vs. Arthroscopic-assisted reduction and internal fixation (ARIF) for tibial plateau fractures

Outcome classification	Traditional surgery group (n = 65)	Arthroscopic-assisted group (n = 68)	χ^2^ value	p-value
Excellent (n)	19	30	–	–
Good (n)	24	28	–	–
Fair (n)	9	3	–	–
Poor (n)	13	7	–	–
Effectiveness rate [% (n/N)]	66.2% (43/65)	85.3% (58/68)	5.657	< 0.05

*n* number of patients, *N* total number of patients in each group, *χ*^*2*^ chi-square statistic

### Postoperative complications of traditional vs. arthroscopic-assisted surgery for tibial plateau fractures

The incidence of postoperative complications was lower in the arthroscopic-assisted group compared to the traditional surgery group. Specifically, traumatic arthritis occurred in 3 of 68 patients (4.4%) in the arthroscopic-assisted group, significantly less than in the traditional group, where 11 of 65 patients (17.0%) were affected (χ^2^ = 4.275, p < 0.05). Similarly, the incidence of wound infection was lower in the arthroscopic-assisted group (4 of 68, 5.9%) than in the traditional group (13 of 65, 20.0%), with a statistically significant difference (χ^2^ = 4.743, p < 0.05). In contrast, there were no significant differences between groups regarding joint stiffness or poor fracture healing. Joint stiffness was observed in 2 of 68 patients (2.9%) in the arthroscopic-assisted group and in 3 of 65 patients (4.6%) in the traditional group (χ^2^ = 0.002, p = 0.959). Poor healing occurred in 3 patients (4.4%) in the arthroscopic-assisted group and in 4 patients (6.2%) in the traditional group (χ^2^ = 0.004, p = 0.951) (Table 5). These findings suggest that arthroscopic-assisted reduction and internal fixation may lower the risk of specific postoperative complications, particularly traumatic arthritis and wound infection, thereby contributing to a safer recovery profile compared with traditional open techniques.

**Table 5:** Comparison of postoperative complications in traditional surgery vs. Arthroscopic-assisted reduction and internal fixation (ARIF) for tibial plateau fractures

Complications	Traditional surgery group (n = 65)	Arthroscopic-assisted group (n = 68)	Χ^2^ value	p-value
Traumatic arthritis (n, %)	11 (17.0%)	3 (4.4%)	4.275	< 0.05
Joint stiffness (n, %)	3 (4.6%)	2 (2.9%)	0.002	0.959
Poor healing (n, %)	4 (6.2%)	3 (4.4%)	0.004	0.951
Wound infection (n, %)	13 (20.0%)	4 (5.9%)	4.743	< 0.05

*n* number of patients, *%* percentage, *χ*^*2*^ chi-square value

### Post-hoc power analysis

A post-hoc power analysis was performed for the primary outcomes, which included surgical metrics (intraoperative blood loss, surgery duration, and incision length) and recovery-related indicators (time to ambulation, fracture healing time, and wound healing time). The power analysis was conducted using G*Power, assuming a medium effect size (Cohen’s d = 0.5) for most outcomes and a significance level of 0.05. With a total sample size of 133 participants (65 in the traditional surgery group and 68 in the arthroscopic-assisted group), the power analysis revealed that the statistical power for all outcomes exceeded 80%, with most outcomes achieving 100% power. Specifically, the power for intraoperative blood loss, surgery duration, and incision length was 100%, while recovery-related outcomes, such as time to ambulation (Cohen’s d = 0.8), fracture healing time (Cohen’s d = 0.8), and wound healing time (Cohen’s d = 0.8), also demonstrated 100% power. These results indicate that the sample size in this study is more than sufficient to detect the significant differences observed, confirming the robustness and reliability of the study’s findings.

## Discussion

Tibial plateau fractures present significant challenges in orthopedic surgery, requiring effective restoration of joint stability and function while minimizing complications and long-term morbidity [18, 19]. This study provides a robust comparison of arthroscopic-assisted versus traditional ORIF for tibial plateau fractures, emphasizing surgical outcomes, recovery times, and complication rates. The novelty of this research lies in its large cohort and comprehensive assessment of surgical efficiency, functional outcomes, and complication rates. Our findings demonstrate that arthroscopic-assisted surgery offers a less invasive alternative to ORIF, with advantages including reduced intraoperative blood loss, shorter surgery duration, smaller incisions, and faster recovery, all while maintaining fracture healing. Clinically, the reduced incidence of traumatic arthritis and wound infections in the arthroscopic group supports its potential to improve patient outcomes, particularly in minimizing long-term joint morbidity. Additionally, superior functional outcomes, including enhanced range of motion and reduced pain, suggest that arthroscopic-assisted surgery facilitates quicker recovery and a return to pre-injury activity levels, thereby improving quality of life [20, 21]. This study highlights the clinical value of ARIF, especially for patients requiring minimally invasive surgery due to comorbidities or the need for rapid recovery. With proper surgical expertise, this approach could become the preferred method for managing tibial plateau fractures, offering an optimal balance of efficiency, recovery, and complication prevention, ultimately enhancing patient care and reducing healthcare costs associated with prolonged recovery and complications [22].

Arthroscopic-assisted surgery has been associated with a lower incidence of postoperative complications, such as traumatic arthritis and wound infection, which can largely be attributed to its minimally invasive characteristics. In contrast to traditional ORIF, which typically requires extensive soft tissue dissection and may compromise the periosteal blood supply, arthroscopic-assisted techniques employ smaller incisions that substantially reduce soft tissue trauma [23, 24]. Preservation of the local biological environment surrounding the fracture may attenuate the postoperative inflammatory response, thereby decreasing the risk of infection and post-traumatic arthritic changes. In addition, arthroscopy provides enhanced, magnified visualization of the intra-articular structures, allowing for thorough debridement and accurate assessment of fracture morphology. This improved visualization facilitates precise fracture reduction and restoration of articular congruity, which are critical for optimizing joint function and minimizing the risk of long-term complications, including joint stiffness and degenerative changes [14, 25].

The reduced postoperative pain and earlier ambulation observed in the arthroscopic-assisted group can be attributed to the limited soft tissue trauma inherent to this minimally invasive approach. Reduced surgical disruption alleviates postoperative pain, which not only improves patient comfort but also facilitates earlier initiation of rehabilitation and ambulation. Early mobilization plays a critical role in orthopedic recovery by preventing muscle atrophy and reducing the risk of joint stiffness, thereby contributing to superior functional outcomes. The greater improvements in postoperative knee extension and flexion observed in the arthroscopic-assisted group further underscore the technical advantages of this approach [26, 27]. Arthroscopic visualization allows precise management of intra-articular fragments and more accurate restoration of the articular surface, which are essential for optimizing knee biomechanics and functional recovery [28, 29]. These benefits are particularly important for long-term joint function, as they support a return to pre-injury activity levels with minimal residual impairment. In addition, the significantly lower postoperative VAS scores in the arthroscopic-assisted group reinforce the association between minimally invasive surgical techniques and reduced postoperative pain [30, 31]. Lower pain levels are closely linked to accelerated rehabilitation, improved patient compliance with physiotherapy, and enhanced overall quality of life, ultimately contributing to more favorable clinical outcomes. Patients in the arthroscopic-assisted group demonstrated higher proportions of excellent and good outcomes than those in the traditional surgery group, which may be partly attributable to the minimally invasive nature of the procedure. Enhanced patient satisfaction may also be influenced by preoperative perceptions and expectations associated with arthroscopic techniques. Previous studies have shown that patients’ expectations before surgery can significantly affect their subjective evaluation of postoperative outcomes and overall satisfaction [32, 33]. Awareness of undergoing a less invasive procedure with the potential for faster recovery may positively shape postoperative perceptions, thereby contributing to improved patient-reported satisfaction and perceived quality of life.

Tay et al. conducted a systematic review and meta-analysis comparing ARIF with traditional ORIF for tibial plateau fractures, finding no significant differences in clinical outcomes or complication rates. They concluded that ARIF is a reliable surgical option but did not establish clear superiority over ORIF. In contrast, our study demonstrates significant advantages of ARIF, particularly in reduced intraoperative blood loss, shorter surgical durations, faster recovery, and improved postoperative mobility. Our larger cohort and detailed recovery metrics further highlight ARIF's clinical benefits in comparison to ORIF. Jiang et al. conducted a meta-analysis comparing ARIF with ORIF for tibial plateau fractures, showing that ARIF resulted in better clinical function, shorter hospital stays, and the ability to identify more intra-articular lesions. However, radiological evaluations and complications did not differ significantly between the two techniques. Our study agrees with Jiang et al. regarding the superior recovery with ARIF, but extends the findings by presenting a broader set of surgical metrics, including incision size and blood loss, as well as the clinical relevance of reduced complications like traumatic arthritis and wound infections. These additional data support ARIF's benefits in improving functional outcomes and minimizing postoperative complications. Nguyen et al. reviewed the outcomes of arthroscopic-assisted tibial plateau fixation (AATPF) for lateral fractures, showing better range of motion, reduced blood loss, shorter hospital stays, and improved functional scores compared to ORIF. Our study corroborates Nguyen et al.’s findings for lateral fractures, but extends the scope by including a broader range of tibial plateau fracture types (Schatzker I-IV) and providing detailed comparisons of intraoperative and postoperative recovery times. Our findings further emphasize ARIF’s clinical advantages, including reduced pain levels and faster overall recovery across a more diverse patient cohort.

### Limitations and future research

Several limitations must be acknowledged. The non-randomized design and potential selection bias may affect the assignment of surgical techniques, influencing outcomes. Future randomized controlled trials (RCTs) with larger, diverse populations are needed to reduce bias and enhance generalizability. The follow-up period was insufficient to assess long-term complications, such as post-traumatic osteoarthritis. Extended follow-up studies are required to evaluate long-term recovery and chronic complications, with patient-reported outcome measures (PROMs) providing deeper insights into the patient-centered benefits of each technique. A formal cost-effectiveness analysis was not included, which is essential for understanding the broader clinical utility of ARIF. Future studies should assess direct and indirect costs, as well as potential savings from reduced complications and faster recovery. While a highly experienced surgical team performed all procedures, the learning curve associated with ARIF may limit the applicability of these findings in less experienced settings. This study was conducted at a single institution, which may limit its generalizability. Multicenter studies with diverse populations and surgical protocols are needed to confirm the external validity of these findings. The exclusion of patients with open tibial plateau fractures and Schatzker V–VI fractures may further limit generalizability. Future research should include these subgroups to assess the applicability of ARIF in managing more complex fractures.

## Conclusions

Comparative analysis suggests that ARIF may offer superior clinical efficacy in the treatment of tibial plateau fractures compared to traditional ORIF. ARIF appears to improve therapeutic outcomes, reduce complications, and enhance safety, potentially making it a preferable approach for managing these fractures.

## Data Availability

The data sets generated and analyzed during this study are not public, but under reasonable requirements, the correspondence author can provide.
